# A systemic review of facial expression recognition (FER) in stroke: diagnosis and emerging applications in rehabilitation

**DOI:** 10.3389/fneur.2026.1744257

**Published:** 2026-05-21

**Authors:** Ruolin Li, Yujia Jin, Jialu Li, Yongxia Mei, Weihong Zhang, Peilin Liu, Zhenxiang Zhang, Beilei Lin

**Affiliations:** 1 School of Nursing and Health, Zhengzhou University, Zhengzhou, Henan, China; 2 Department of Neurology, Henan Huaxian People’s Hospital, Anyang, China

**Keywords:** diagnosis, facial expression recognition, systemic review, rehabilitation, stroke

## Abstract

**Background:**

Stroke remains a leading cause of long-term disability and mortality worldwide, the early identification and evaluation of rehabilitation effectiveness are of great significance. Facial expression recognition (FER), a computer vision technology, offers potential for early screening and monitoring in many conditions. However, its application in stroke remains underexplored.

**Aims:**

To offer insights into how the FER can be used in stroke identification and rehabilitation monitoring.

**Methods:**

We systematically searched four databases, including PubMed, Web of Science, CNKI, and WanFang, for studies on FER applications in stroke, from database inception to January 2025.

**Results:**

A total of 1,855 studies were identified, of which nine met the inclusion criteria (e.g., 8 diagnostic studies and 1 rehabilitation trial). Eight studies demonstrated FER’s diagnostic utility for stroke, achieving accuracies ranging from 82 to 98% through facial asymmetry analysis during standardized tasks in public/private datasets. Specific tasks such as KISS, SPREAD, and non-speech movements were particularly effective. One study achieved 99.81% accuracy in monitoring rehabilitation intensity by classifying real-time facial expressions to tailor training intensity. The data sources included images (66.7%) and clinical patients database (55.6%).

**Conclusion:**

FER technology exhibits substantial potential as an auxiliary tool in stroke diagnosis and emerging rehabilitation applications by enabling precise analysis of mandibular and facial movements. Nevertheless, FER models face considerable challenges in real-world clinical translation. Future research could integrate multimodal data and in-the-wild databases to facilitate the clinical implementation of FER technology, thereby improving care delivery for both patients and clinicians and reducing patient mortality and disability.

## 1 Introduction

Stroke has become a significant global health challenge, with an estimated 6.5 million deaths annually, a figure anticipated to increase to approximately 8 million by 2030 (1). Early diagnosis and intervention are critical for reducing morbidity and mortality, as the efficacy of treatment diminishes with delayed detection (2). The gold-standard diagnostic clinical history, physical examination, and neuroimaging (CT/MRI) (3). Despite these core diagnostic strategies, a number of supplementary stroke identification tools have been integrated into routine clinical practice. Research indicates that scales including the Cincinnati Pre-hospital Stroke Scale, the Face Arm Speech Test, and the Recognition of Stroke in the Emergency Room (ROSIER) yield sensitivities between 83 and 91%, yet their specificity remains comparatively limited (4). However, these methods often fail to capture subtle neurological signs such as facial asymmetry—a key indicator of stroke—related cranial nerve impairment. Facial asymmetry serves as one of the early clinical manifestations in stroke patients (3), and its timely recognition may facilitate early diagnosis and intervention, which are pivotal for mitigating morbidity and mortality, as each hour of treatment delay results in the loss of 1.9 million neurons (5). Nevertheless, challenges such as delays in medical care (6), missed diagnoses (7), and misdiagnoses (7) frequently hinder timely stroke management, worsening patient outcomes (8). Although current diagnostic approaches provide valuable tools, they often lack the timeliness and accuracy required for acute stroke settings. These challenges underscore the urgent need for more accurate and rapid diagnosis.

Due to the challenges in traditional stroke diagnosis (9), alternative approaches such as facial images have gained attention (10). Rehman (11) employed a hybrid deep learning model to predict the risk of early-stage brain stroke. In general, two primary technical strategies are widely adopted for facial image-based stroke evaluation. Landmark-dependent geometric methods track key facial feature points (e.g., mouth corners, eyebrows) to quantify facial asymmetry and abnormal mobility, which is consistent with clinical manifestations of facial weakness in stroke patients (12, 13). By comparison, deep learning—based approaches directly extract patterns of emotion and facial movement from facial images. Although such methods frequently achieve higher recognition accuracy, they generally demand larger training datasets and more substantial computational resources (14). FER conveys emotions, and affective states to the external world through muscle movements, eye behaviors, and eyebrow actions (15, 16). It has been widely used, such as depression diagnosis (17), autism spectrum disorder identification (18), and it is significantly improving care quality (19). Following the onset of stroke, patients’ impaired facial nerve function, including unilateral facial paralysis (facial palsy), drooping of the mouth corner, and loss of the nasolabial fold, leading to characteristic changes in facial expressions (3). The visual nature of these signs makes FER technology a promising tool for early stroke detection.

Moreover, FER has shown promise in stroke rehabilitation. Rehabilitation plays a critical role in improving the quality of life for stroke survivors (20). Functional recovery in stroke rehabilitation requires sensitive tracking of neurological improvements, yet conventional assessment tools (e.g., clinical scales) suffer from subjectivity and low resolution for detecting early-stage changes (21, 22). A preliminary study explored the application of Emotional AI-driven FER in the context of tele-rehabilitation, allowing therapists to adjust exercise intensity, provide encouragement, or identify when a patient is struggling, ultimately aiming to improve the quality and effectiveness of remote care and patient outcomes (23). Therefore, FER can address rehabilitation monitoring by providing millimeter-level measurements of facial muscle symmetry during dynamic expressions, enabling data-driven rehabilitation adjustment (23, 24). Through automated analysis of facial muscle symmetry and dynamic expression patterns, FER might enable several key functions: early detection of neurorehabilitation progress, real-time feedback during therapeutic exercises, and data-driven adjustment of rehabilitation protocols.

In summary, FER can detect characteristic facial muscle impairments (e.g., facial weakness, facial paresis, and eye movement abnormality) of stroke patients (25). These impairments can also affect specific facial regions, manifesting as asymmetrical drooping during natural expressions (26). By analyzing the millimeter-level facial changes, FER might support stroke diagnosis and rehabilitation. Despite the growing utilization of FER in neurological research (11), no systematic review has comprehensively examined the scope, clinical applications, and implementation challenges specially for stroke. Therefore, the purpose of this review is to synthesize the diagnostic utility of FER technology in stroke populations, composite its rehabilitative applications, and identify methodological limitations and challenges in current studies.

## 2 Methods

### 2.1 Search strategy

We systematically searched both English and Chinese electronic databases (PubMed, Web of Science, CNKI, and WanFang) for relevant articles published from inception to January 2025. The search strategy utilized the following terms: (TS = (facial expression OR facial recognition OR emotion recognition)) AND TS = (stroke OR cerebrovascular accident OR apoplexy OR brain vascular accident OR vascular accident brain OR cerebral hemorrhage OR cerebral infarction OR post-stroke OR cerebral ischemia OR hemorrhage stroke OR ischemic stroke OR acute ischemic stroke OR cerebrovascular disorders). The search terms were designed by the research team with excellent experience in doing systematic reviews. After data screening, one reviewer manually screened the reference lists of all included studies to identify additional relevant articles; the second reviewer checked and approved the results. Records were independently assessed by two reviewers to predefined eligibility criteria. When disagreement arose, a third author helped to reach a consensus.

### 2.2 Eligibility criteria

Studies were selected based on the following inclusion criteria: (1) Studies involving human stroke patients diagnosed according to internationally recognized criteria (e.g., WHO ICD-11, AHA/ASA guideline), or utilizing facial images/videos directly acquired from stroke patients; (2) Intervention/Technology: studies applying FER technology to analyze stroke patients’ facial expressions; (3) Outcome: studies quantifying facial muscle movements (e.g., asymmetry scores, AUs intensity) during spontaneous expressions or standardized emotion elicitation tasks (e.g., smiling, eyebrow raising); (4) Study Design: original empirical studies; (5) Language: articles in English or Chinese. Exclusion criteria included: (1) Studies focusing on stroke patients’ ability to recognize other’ emotions (e.g., emotion perception tasks); (2) Non-original research (e.g., reviews, editorials, case reports); (3) Animal studies or non-human data.

### 2.3 Data extraction

A standardized data extraction form was developed in Microsoft Excel to systematically record the following information from each included study. The extraction form was iteratively revised based on feedback from domain experts (e.g., neurologists and AI specialists) to ensure clinical and technical relevance. One reviewer (RLL) independently extracted the following data: first author, year of publication, country of origin, study objectives, study design, data resources, emotions or standardized tasks, participants, data type (sample size), outcomes, results and application context. The second reviewer (YJJ) checked all the data. Discrepancies were resolved by discussion within the research group except for PLL and WHZ.

## 3 Results

**Figure 1:**
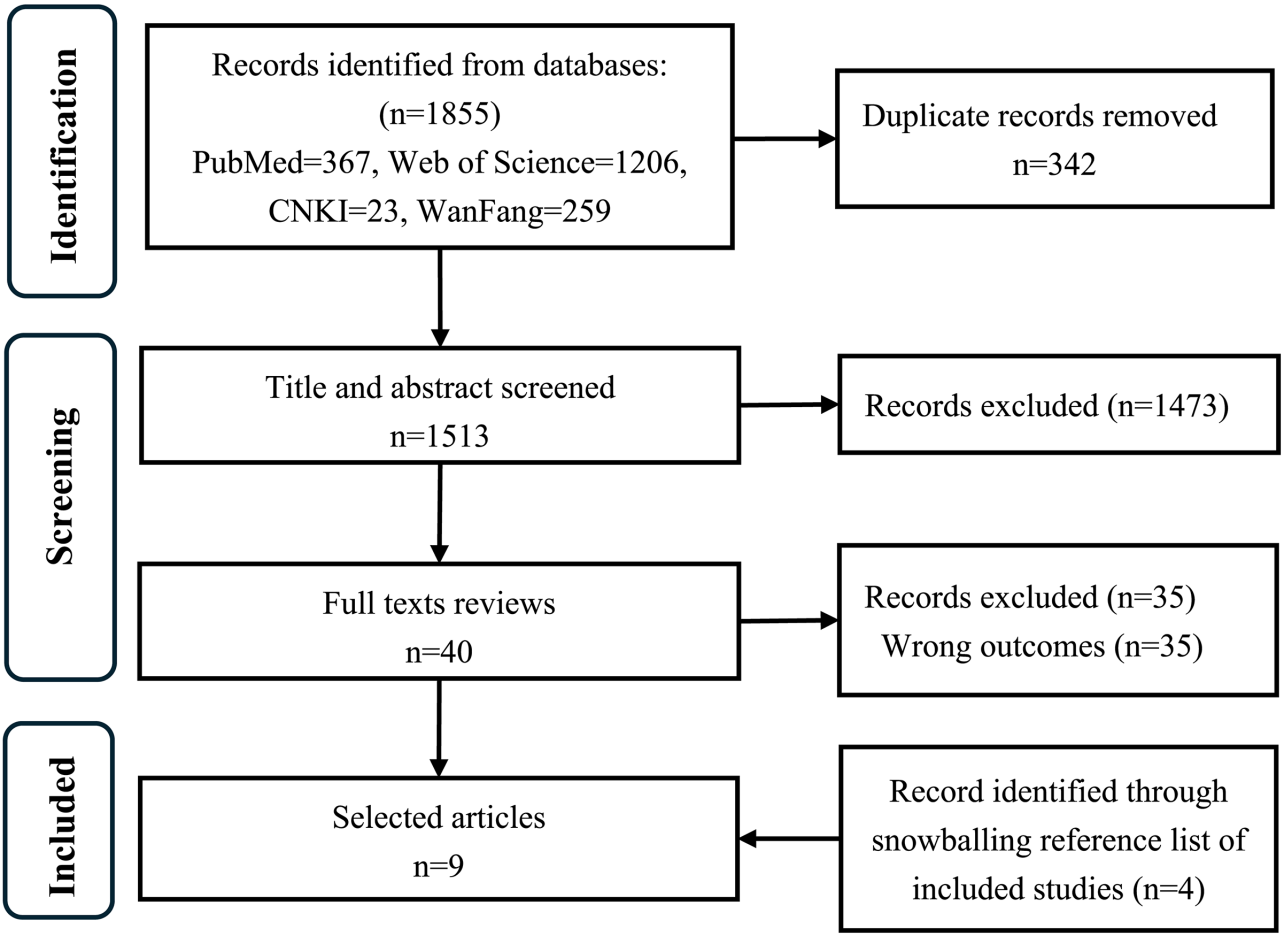
Flow diagram of literature screen.

A total of 1,855 records were retrieved. After duplicate removal, 1,513 studies underwent title and abstract screening, resulting in the exclusion of 1,473 article. Full texts and references of 40 articles were examined, and four additional citations were identified through reference list screening. Finally, nine met the inclusion criteria and were included in the review. The literature screening process is shown in Figure 1.

### 3.1 Characteristics of included studies

**Figure 2:**
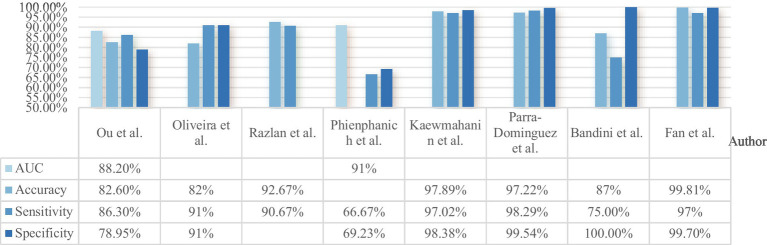
Performance comparison of stroke facial recognition models from different studies. Only the best performance reported in each study is presented.

The nine articles, published between 2011 and 2025, originated from seven countries (e.g., Brazil, China, Malaysia, Thailand, Mexico, Canada, Switzerland). With the exception of study (27) which additionally included facial expression data from patients with facial paralysis, the other eight studies (12, 13, 28–33) analyzed facial expression information in both stroke patients and healthy participants. The study samples were categorized into two types: individuals and images. Among the included nine studies, three utilized only individuals (30, 31, 33), four employed solely images (12, 27, 29, 32), while two incorporated both individuals and images (13, 28). Specifically, four studies used only a publicly available dataset (12, 27, 29, 32), one study used both a public dataset and established its own database (13), whereas four study established its own proprietary database (28, 30, 31, 33). The sample sizes ranged from 25 to 253, while the number of analyzed images varied between 480 and 36,500. The detailed information is shown in Table 1 and Figure 2.

**Table 1:** Characteristics of included studies (*n* = 9).

Author (Year)	Country	Objectives	Study design	Data resources	Emotions/Standardized tasks	Participants	Data type (sample size)	Outcomes	Results	Application context
Ou et al. (2025) (33)	China	To assess early stroke.	Cross-sectional study	Private dataset	1)Emotions:N/A; 2)Standardized tasks: N/A	1)Stroke patients; 2)Healthy people	Participants (253)	AUC, accuracy, F1 score	1)AUC: 0.882; 2)Accuracy: 82.6%; 3)Sensitivity:86.3%; 4)Specificity:78.95%.	Diagnosis
Oliveira et al. (2024) (12)	Brazil	To develop a diagnostic model for stroke patients.	Case-control study	TNF	1)Emotion: N/A; 2)Standardized tasks: KISS, OPEN, SPREAD, PA, PATAKA, BBP, and BLOW;	1)Stroke patients; 2)Healthy people	Videos (168)	Accuracy, sensitivity, specificity and Log-Likelihood Ratio;	1)Accuracy: 82%; 2)Sensitivity:91%; 3)Specificity: 91%; 4)Best differentiate: KISS and SPREAD.	Diagnosis
Razlan et al. (2024) (32)	Malaysia	To distinguish between normal and stroke paralyzed.	Retrospective study	The UTK Face Cropped dataset, & the Facial Droop and Facial Paralysis dataset	1)Emotions: N/A; 2)Standardized tasks: N/A	1)Stroke patients; 2)Healthy people	Images (2,000)	Accuracy, precision, recall, F1 score;	1)Accuracy: 92.67%; 2)Sensitivity:90.67%.	Diagnosis
Phienphanich et al. (2023) (28)	Thailand	To develop an accurate diagnosis model for stroke.	Case-control study	FaceGAN, & private dataset	1)Emotions: neutral and smiling; 2)Standardized tasks: N/A;	1)Stroke patients; 2)Healthy people	Images (28,800) and participants (63)	Sensitivity, specificity, Cohen's kappa, F1 score, Area Under the Curve;	1)AUC: 0.91; 2)Sensitivity: 66.67%; 3)Specificity: 69.23%.	Diagnosis
Kaewmahanin et al. (2022) (29)	Thailand	1)To differentiate between stroke patients and healthy people; 2)To use cosine similarity for stroke detection.	Case-control study	TNF	1)Emotion: N/A; 2)Standardized tasks: KISS, OPEN, BIGSMILE, SPREAD, BROW, and BLOW;	1)Stroke patients; 2)Healthy people	Images (496)	Accuracy, sensitivity, specificity and F1 score of the classification;	1)Accuracy: 97.89%; 2)Sensitivity: 97.02%; 3)Specificity: 98.38%; 4)Best differentiate: low lip and eyebrow.	Diagnosis
Parra-Dominguez et al. (2021) (27)	Mexico	1)To identify asymmetry levels between the two sides of the face; 2)To identify stroke patients.	Case-control study	TNF, & MEEI	1)Emotion: N/A; 2)Standardized tasks: at rest, eyebrow elevation, light effort eye closure, full effort closure, light effort smile, full effort smile, pucker, lip depression, KISS, OPEN, SPREAD, PA, PATAKA, BBP, BLOW, BROW, and BIGSMILE;	1)Stroke patients; 2)Healthy people; 3)Others	Images (1516)	Accuracy, sensitivity, specificity and precision;	1)Accuracy: 97.22%; 2)Sensitivity: 98.29%; 3)Specificity: 99.54%.	Diagnosis
Bandini et al. (2018) (30)	Canada	To discriminate patients with facial impairment due to stroke.	Case-control study	Private dataset	1)Emotion: N/A; 2)Standardized tasks: rest, OPEN, KISS, BLOW, SPREAD, PA, PATAKA, and BBP;	1)Stroke patients; 2)Healthy people	Participants (23)	Accuracy, sensitivity, specificity;	1)Accuracy: 87%; 2)Sensitivity: 75.0%; 3)Specificity: 100.0%; 4)Best differentiate: BBP, BLOW, and SPREAD;	Diagnosis
Schimmel et al. (2011) (31)	Switzerland	To quantify facial muscle function in stroke patients.	Case-control study	Private dataset	1)Emotions: rest, force, pose, and Ebl; 2)Standardized tasks: N/A	1)Stroke patients; 2)Healthy people	Participants (27)	Absolute side differences, offline measurement of distances between facial landmarks, and House-Brackmann scale grades;	1)Accuracy: N/A; 2)Best differentiate: the lower facial muscles; 3)Others: potential to detect facial paralysis in patients;	Diagnosis
Fan et al. (2022) (13)	China	To determine rehabilitation intensity.	Cross-sectional study	FER+, & RAF - DB, & private dataset	1)Emotions: happy, sad, surprised, angry, fearful, disgusted, neutral, contempt, painful, strained, and tired; 2)Standardized tasks: N/A;	1)Stroke patients; 2)Healthy people	Images (36,500) and participants (42)	Accuracy, params and GFLOPs;	1)Accuracy: 99.81%; 2)Sensitivity: 97%; 3)Specificity: 99.7%.	Rehabilitation

TNF dataset, Toronto Neuroface dataset; FER+ dataset, The Face Expression Recognition Plus dataset; RAF-DB,The Real-world Affective Faces Database; MEEI database, the Massachusetts Eye and Ear Infirmary database; FaceGAN, FaceGAN dataset, KISS, pretending to kiss a baby; OPEN, maximum opening of the jaw; SPREAD, pretending to smile with tight lips; PA,reproductions of the syllable /pa/ as quickly as possible on a single breath; PATAKA, speaking of the word /pataka/ as fast as possible on a single breath; BBP, speaking the sentence “Buy Bobby a Puppy” at a comfortable speaking rate and intensity; BLOW, pretending to blow a candle; BIGSMILE, making a big smile; BROW, repetitions of raising the eyebrows; rest, face at rest; force, aggressive smile; pose, posed smile; Ebl, maximum lifted eyebrow; Aus, Action Units; LLR, Log-Likelihood Ratio; N/A, none; Given the primary objective of this scoping review is to synthesize the application potential of FER technology in the field of stroke, the table only presents the optimal performance metrics (including AUC, accuracy, specificity, and sensitivity) of FER technology reported in the included studies.

### 3.2 Applications of FER

#### 3.2.1 FER assisted diagnosis

Eight studies (12, 27–33) demonstrated the potential of FER technology as an adjunct tool for stroke diagnosis. These studies utilized facial features analysis, derived from either database images/videos (12, 13, 27–29, 32) or prospectively collected facial expressions from stroke patients and healthy people (13, 28, 30, 31, 33), to differentiate stroke from other conditions (e.g., health or other diseases).

##### 3.2.1.1 Diagnosis models built on public datasets

A total of four studies developed stroke diagnostic model based on the data from public datasets. The first widely used database is TNF, which was built by Toronto University. The Study found that it is currently the sole publicly available resource with orofacial impairment due to disorders of the nervous system (such as stroke and ALS), together with ground truth facial landmarks and clinical metadata at the same time (34). TNF is a single-center, cross-sectional dataset, consisting of 261 facial expression videos collected from 36 people, including 11 healthy individuals, 14 stroke patients, and 11 people with Amyotrophic Lateral Sclerosis (ALS). Facial expressions were collected based on nine standardized tasks (KISS, OPEN, SPREAD, PA, PATAKA, BBP, BROW, BIGSMILE, and BLOW). Oliveira et al. (12) developed a user-friendly model that facilitated rapid screening of stroke patients, with preliminary validation conducted through a pilot study. They selected dynamic videos from seven tasks (e.g., KISS, OPEN, SPREAD, PA, PATAKA, BBP, and BLOW) in the TNF dataset and analyzed data using the Python Facial Expression Analysis Toolbox to identify and quantify facial muscle movements. The results indicated that, when participants performed KISS and SPREAD tasks, the model achieved 82% accuracy in distinguishing stroke patients from healthy controls; for all other tasks, accuracy dropped below 77%, which may be attributed to the complexity of video analysis. It concluded that the lower facial regions demonstrated greater suitability for stroke patient recognition compared to the upper facial regions.

Similar to the study by Oliveira et al. (12), Kaewmahanin et al. (29) analyzed data from the TNF dataset to differentiate between stroke patients and healthy people using cosine similarity, a method that measures vector similarity in high-dimensional space. All images at the peak action of six non-speech tasks (KISS, OPEN, SPREAD, BROW, BIGSMILE, and BLOW). For each facial side, 29 facial landmarks were identified, and vectors between these points and a nasal reference point were computed. Left-side vectors were flipped to enable asymmetry analysis via cosine similarity comparisons with right-side vectors. Finally, the overall accuracy reached 97.89% across all tasks, while the accuracy for individual subtasks ranged from 98.2 to 99.57%. It was suggested that compared to other frames in a dynamic sequence, the video frame at the peak of an action is more effective at capturing the muscular changes associated with different facial expressions. Moreover, it was found that facial weakness in stroke patients is usually found around the eye line and mouth line since the study employed non-speech tasks. The findings suggested the lower lip and the eyebrows constitute the primary visual foci for accurate recognition, in stroke individuals.

Moreover, due to the limited availability of stroke-specific databases, TNF and other databases that included multiple conditions (e.g., facial paralysis) or healthy populations were utilized for diagnostic model development and validation. Parra-Dominguez et al. (27) incorporated the TNF and MEEI database, which comprised 10 healthy subjects and 50 paralysis patients (i.e., 25 suffering from flaccid and 25 from nonflaccid paralysis), to identify asymmetry levels for detecting stroke patients. The analysis focused on 51 key facial points (including eyebrows, eyes, nose, and mouth). Facial asymmetry in patients was quantified by calculating inter-landmark distances after performing tilt correction to adjust for symmetry deviations. The results demonstrated that the proposed classification model achieved an average accuracy of 94.06% for stroke patients in the MEEI database, with comparable performance (97.22%) on the TNF dataset. It was suggested that, compared with other models, the classification model in this study is capable of identifying any stroke patients’ facial expression, thereby avoiding misdiagnoses that may arise when patients are unable to perform certain specified actions due to their illness.

Similarly, Razlan et al. (32) employed non-specialized stroke databases to develop a deep learning-based facial paralysis detection system capable of accurately identifying facial paralysis from real-time webcam feeds and static images. They utilized 2,000 images of facial paralysis patients caused by stroke and healthy faces from Kaggle, including the UTK Face Cropped dataset, which encompasses diverse ages, genders, and ethnicities, and the Facial Droop and Facial Paralysis dataset, containing cases of Bell’s palsy, stroke, and brain tumors. These images were divided into training, validation, and testing sets, with facial images processed using InceptionResNetV2, achieving 92.7% accuracy in distinguishing normal and paralyzed facial expressions. It was suggested that real-time webcam and static images could facilitate continuous monitoring of stroke patients’ condition, thereby providing valuable support for healthcare professionals to adjust treatment plans in a timely manner and maximize therapeutic outcomes.

##### 3.2.1.2 Diagnosis model based on private databases

Except for the development of diagnostic models based on public datasets, three studies collected facial emotions from clinical stroke patients and developed private databases (30, 31, 33). Compared to public databases, the use of clinical data enables simulation of real-world clinical scenarios by accounting for practical challenges such as variable lighting conditions and head tilt. In 2011, a research team from Switzerland (31) evaluated facial feature vectors of participants to assess facial muscle function in stroke patients. They recruited 27 stroke patients. All participants performed three facial expressions from standardized tasks (force, aggressive smile; pose, posed smile; Ebl, maximum lifted eyebrow; rest, face at rest). Both the affected and non-affected sides were analyzed, and facial asymmetry was quantified by calculating the absolute differences between the two sides. These distance-based measurements were then used to assess the severity of facial impairment in participants, enabling the discrimination of stroke patients and evaluation of their facial muscle function. The results demonstrated that the 3D system showed high sensitivity in evaluating facial muscle function in stroke patients. Similarly, a study found stroke lesions had a more pronounced impact on lower facial musculature than on upper regions. Furthermore, quantitative assessment of the differential impairment between upper and lower facial regions may provide clinicians with valuable data to support distinguishing central from peripheral facial palsy.

Furthermore a research team from Canada (30) used 3D coordinates to assess facial impairment in stroke in 2018. They enrolled 12 stroke patients and 11 healthy people, who all performed eight tasks (rest, KISS, OPEN, SPREAD, PA, PATAKA, BBP, and BLOW). Subsequently, facial asymmetry in stroke patients was assessed by extracting geometric features and analyzing motion trajectories across 184 video recordings. The results indicated that the video-based automated monitoring system achieved an accuracy ranging from 56.5 to 87% in distinguishing stroke patients from healthy individuals, markedly below the performance reported in other studies. This discrepancy is probably attributable to under-fitting caused by the limited sample size. Meanwhile, this study found that tasks involving lower-facial muscular activity (e.g., BBP, BLOW, and SPREAD) demonstrated the highest discriminatory power for identifying stroke patients.

Building on these efforts (30, 31), Ou et al. (33) developed a multi-modal model for early stroke diagnosis by integrating facial movement analysis with motor function assessment in 2025. They enrolled 132 stroke patients and 121 healthy people. Based on the FAST (Face-Arm-Speech-Time) protocol, the researchers captured video recordings of facial movements and other motor tasks (e.g., hand lifting, leg lifting, and pointing at the nose) to extract stroke-related action features. Additionally, audio data were collected to assess speech impairments. These multi-modal features were fused to evaluate both cognitive and motor functions, enabling the discrimination between stroke and non-stroke patients, achieving an AUC level of around 80% in the video module and audio module. It suggested that movements and speech had clinical potential in predicting of stroke.

##### 3.2.1.3 Diagnosis model based on mixed data

Only one study incorporated both public and private data sources. A research team from Thailand (28) developed an accurate diagnosis model for stroke patients by employing facial landmarks and affine transformation matrices in 2023. First, the FaceGAN public dataset comprising 14,400 pairs neutral-to-smile and smile-to-neutral facial expression images was used as the training set. Then, they enrolled 12 healthy people, 51 stroke patients with different levels of facial muscle weakness, to collect original images as a validation set. Cosine similarity and a customized loss function were employed to differentiate expressions trajectories between neutral and smiling states. The proposed model demonstrated robust performance, achieving an AUC of 0.76 on the FaceGAN dataset, and 0.91 on the real subjects dataset. In contrast to studies that utilized either exclusively public or private data, the methodology employed by (28) offers a more complete and rigorous evaluation. This two-stage process is a key strength, directly addressing the challenges of clinical translation and resulting in a model whose performance on real patient data demonstrates significant promise for practical application.

#### 3.2.2 FER aided rehabilitation

Of the nine included studies, one study from China has explored the auxiliary role of FER in stroke rehabilitation (13). Fan et al. (13) developed a FER model using a Patch-Convolutional Vision Transformer (FER-PVCT) to classify real-time facial expressions from patients to assess whether the training intensity was appropriate. Facial expression images of healthy subjects were sourced from two public datasets—RAF-DB (containing seven basic emotions: happy, sad, surprised, angry, fearful, disgusted, and neutral) and FER+ (which introduced an additional category for contempt) to develop a diagnostic model. Then, they established a private dataset comprising 37 stroke patients and 5 healthy controls to validate the model’s specificity in clinical setting. This dataset covered four basic emotions (happy, sad, surprised, and angry) and four special emotions (painful, strained, tired, and neutral). The results indicated that the proposed FER-PVCT model achieved high accuracy, attaining over 88% on public datasets and 99.81% on their own dataset. However, the exceptionally high accuracy on the proprietary dataset may be attributed to overfitting because of small sample size. It was suggested that FER technology could monitor patient expressions during rehabilitation to assess exercise intensity, thereby providing valuable data to support clinicians in adjusting rehabilitation training regimens.

## 4 Discussion

The current evidence regarding FER applications in stroke diagnosis and rehabilitation remains preliminary. The nine studies included in this review are predominantly early-stage investigations and validation, including model and algorithm development as well as pilot trials. These findings underscore the potential of FER technology to serve as a valuable adjunct tool in stroke care, particularly for the purpose of diagnosis and rehabilitation monitoring.

This review highlights that FER technology holds substantial value in stroke identification and can alleviate the diagnostic burden on clinical staff. By analyzing abnormalities in patients’ real-time facial muscle movements, FER enables rapid and non-invasive stroke detection and disease severity assessment (35), thereby improving patient outcomes, delaying disease progression, and enhancing overall quality of life (36). In contrast, traditional neurological diagnostic techniques, including neuroimaging and comprehensive motor assessments, are often invasive, costly, and time-intensive (37). As a non-invasive alternative, FER can serve as an adjunct diagnostic tool, facilitating continuous monitoring to optimize treatment strategies and effectively manage clinical symptoms (38). Notably, despite these distinct merits, the clinical translation and large-scale implementation of FER in stroke care remain limited. Most current studies are still in the laboratory or preliminary validation phase, with insufficient exploration of real-world clinical scenarios, standardized protocols, and long-term systems stability (12).

Beyond diagnostic applications, FER is emerging as a promising tool in the field of stroke rehabilitation. Although both applications involve the detection of facial expressions in stroke patients, the underlying principles of FER in the diagnostic and rehabilitation fields are highly similar. Changes in facial expressions during rehabilitation training can reflect patients’ fatigue levels, and FER technology can identify such subtle changes, which can indicate patients’ physical functional status and assist clinicians in making personalized adjustments to the training intensity (39, 40). Additionally, during the rehabilitation process, as the severity of the patient’s condition dynamically changes, their facial muscle dysfunction also evolves accordingly (41). FER technology can achieve dynamic monitoring and evaluation of rehabilitation progress by identifying differences in the standardization of facial expressions in the same patient across different rehabilitation stages. However, only one study has explored the application of FER technology in the rehabilitation field (13). Future research can further extend the application of FER technology to the disease rehabilitation stage, where personalized rehabilitation strategies can be adjusted based on changes in facial expression during the rehabilitation process.

However, the instability of FER models significantly limits their clinical application. Studies utilizing public datasets have achieved relatively high accuracy (82–97.89%), whereas those based on clinical data have demonstrated lower accuracy (56.5–87%) (30). Meanwhile, multimodal approaches have enhanced diagnostic robustness (AUC = 0.882) by integrating more diverse data sources (33). In contrast, within the field of rehabilitation, FER technology has been shown to achieve an accuracy of 99.81% (13). Such substantial fluctuations in accuracy restrict FER technology to the role of an auxiliary tool, which underscores the necessity of high-standard model validation. So future research could integrate multimodal facial expression data (42) to further improve specificity and stability of both diagnostic and rehabilitation application.

Furthermore, reduced lower facial movements have been identified as a predominant feature in distinguishing stroke patients (12, 29, 31). This finding aligns with the well-established clinical observation that facial weakness in hemispheric stroke is typically more prominent in the lower face than in the upper face (43–45). Therefore, further in-depth investigation into the changes affecting the lower facial musculature in stroke patients is warranted. Nevertheless, the accuracy of FER can be significantly affected by multiple factors (46). Misalignment in head position may lead to the loss or displacement of eyebrow, directly impairing the extraction of geometric features. Second, lighting intensity and shadows can alter appearance features in deep learning models and reduce classification accuracy. In addition, standard cameras struggle to capture subtle facial expressions in patients, which may be masked by exaggerated voluntary movements, yet such subtle changes are clinically important for stroke patients. Therefore, addressing these challenges in the future will require in-the-wild datasets, 3D tracking techniques (46), and other approaches, but specific intervention strategies still require more in-depth research by scholars to identify effective solutions.

As an auxiliary diagnostic tool, FER can provide valuable reference data for the preliminary identification and screening of stroke during emergency transport and clinical evaluation. First, guidelines recommend that stroke patients receive treatment as early as possible to delay disease progression (47). FER technology can enable pre-hospital screening of patients by recognizing stroke-induced facial changes associated with the disease (48). Second, inaccurate outpatient triage when patients seek medical care can delay the treatment time window and directly impact prognosis (49). FER technology can serve as an auxiliary triage tool to improve the accuracy of stroke identification and expedite patient treatment (50). Finally, during the rehabilitation stage, FER technology can provide real-time facial monitoring to inform clinicians in adjusting appropriate rehabilitation strategies and expand the range of options for patients’ rehabilitation settings (13). However, the clinical application of FER technology is inevitably confronted with multiple challenges. For medical staff, first, the skepticism of experienced neurologists regarding the acceptability and accuracy of FER technology directly hinders its clinical adoption (51). Second, stroke already has a well-established clinical diagnostic pathway, and whether physicians or nurses have sufficient time to operate the FER system will also influence the frequency of its use (52). For patients, their ability to cooperate in performing designated movements and their concerns about facial privacy are also key factors affecting the clinical application of FER technology (53). Future research should optimize the performance of FER technology, reduce user requirements, improve the accuracy and usability of FER systems, and provide accurate and rapid assessment results for patients with suspected stroke.

## 5 Limitations

Although our comprehensive search strategy and rigorous screening process ensured the systematic identification of relevant studies on FER applications in stroke care, this review has several limitations. The relatively small number of included studies restricts the generalizability and external validity of our conclusions. Additionally, the potential for publication bias in the existing literature may influence the perceived efficacy of FER technologies, as positive results are more likely to be published. The lack of meta-analytic data further precluded a quantitative synthesis of results, limiting our ability to draw robust statistical conclusions regarding the overall clinical impact. Therefore, while this review provides valuable conceptual insights and identifies key themes for future research, its findings should be interpreted with caution due to these methodological constraints.

## 6 Conclusion

In conclusion, this review synthesizes evidence that firmly supports the potential of FER as a transformative, non-invasive tool spanning from stroke diagnosis to emerging rehabilitation applications. FER technology offers valuable quantitative auxiliary diagnostic indicators by analyzing changes in lower facial asymmetry and enables real-time monitoring of patient engagement during rehabilitation. However, the clinical application of FER technology remains limited and is constrained by multiple factors, including lighting conditions and facial angles, with unstable model accuracy. Future research can adopt in-the-wild datasets and apply FER technology to clinical scenarios such as pre-hospital screening, clinical diagnosis, and remote rehabilitation of stroke patients, thereby improving the efficiency of stroke patients’ access to correct treatment.
